# QuickStats

**Published:** 2015-08-14

**Authors:** FROM THE NATIONAL CENTER FOR HEALTH STATISTICS

**Figure:**
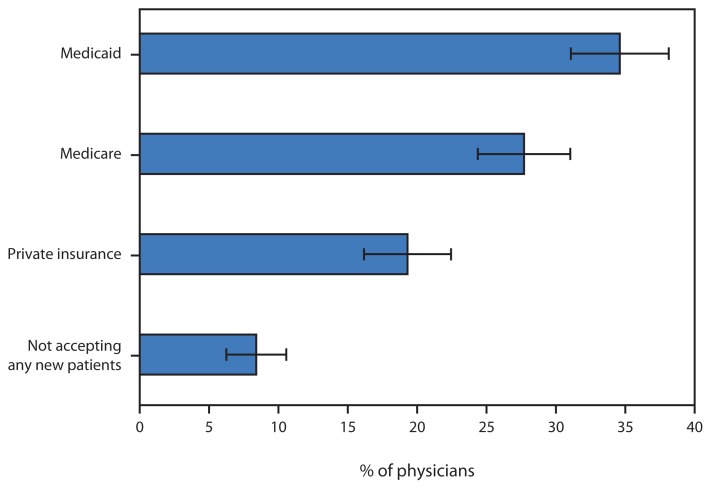
Percentage* of Office-Based Primary Care Physicians Not Accepting New Patients, by Source of Payment — United States, 2013 * With 95% confidence intervals.

In 2013, overall, 8.4% of primary care physicians reported that they did not accept new patients. However, acceptance varied by the patient’s expected payment source: 35% of physicians did not accept new Medicaid patients, 27.7% did not accept new Medicare patients, and 19.3% did not accept new privately insured patients.

**Source:** National Electronic Health Records Survey data, available at http://www.cdc.gov/nchs/ahcd/ahcd_questionnaires.htm.

**Reported by:** Esther Hing, MPH, ehing@cdc.gov, 301-458-4271; Sandra Decker, PhD; Eric Jamoom, PhD.

